# Dominant Inheritance of Field-Evolved Resistance to *Bt* Corn in *Busseola* *fusca*

**DOI:** 10.1371/journal.pone.0069675

**Published:** 2013-07-02

**Authors:** Pascal Campagne, Marlene Kruger, Rémy Pasquet, Bruno Le Ru, Johnnie Van den Berg

**Affiliations:** 1 Unité de Recherche IRD 072, CNRS UPR9034, Laboratoire Evolution, Génome et Spéciation, Gif-sur-yvette, France; 2 Université Paris-Sud 11, Orsay, France; 3 Unit of Environmental Sciences and Management, North-West University, Potchefstroom, South Africa; 4 Noctuid Stem Borers Biodiversity in Africa Project, Environmental Health Division, *icipe* (International Centre of Insect Physiology and Ecology, Nairobi, Kenya; 5 Department of Ecology, Evolution and Natural Resources, Rutgers University, New Brunswick, New Jersey, United States of America; French National Institute for Agricultural Research (INRA), France

## Abstract

Transgenic crops expressing *Bacillus thuringiensis* (*Bt*) toxins have been adopted worldwide, notably in developing countries. In spite of their success in controlling target pests while allowing a substantial reduction of insecticide use, the sustainable control of these pest populations is threatened by the evolution of resistance. The implementation of the “high dose/refuge” strategy for managing insect resistance in transgenic crops aims at delaying the evolution of resistance to *Bt* crops in pest populations by promoting survival of susceptible insects. However, a crucial condition for the “high dose/refuge” strategy to be efficient is that the inheritance of resistance should be functionally recessive. *Busseola* *fusca* developed high levels of resistance to the *Bt* toxin *Cry 1Ab* expressed in *Bt* corn in South Africa. To test whether the inheritance of *B* *. fusca* resistance to the *Bt* toxin could be considered recessive we performed controlled crosses with this pest and evaluated its survival on *Bt* and non-*Bt* corn. Results show that resistance of *B* *. fusca* to *Bt* corn is dominant, which refutes the hypothesis of recessive inheritance. Survival on *Bt* corn was not lower than on non-*Bt* corn for both resistant larvae and the F_1_ progeny from resistant × susceptible parents. Hence, resistance management strategies of *B* *. fusca* to *Bt* corn must address non-recessive resistance.

## Introduction

The role agricultural biotechnologies could play in reducing yield gaps of important crops in the developing world [1] depends on the sustainability of genetically modified crops [2]. Genetically engineered crops such as maize and cotton, expressing insecticidal toxins of *Bacillus thuringiensis* (*Bt*), have been adopted at high rates in both developed and developing countries [3]. In this regard, the questions of ‘how’ and ‘how rapidly’ target pests evolve resistance to crops expressing *Bt* toxins have received fair amount of scientific attention [4]. Field-evolved resistance is defined as a genetically based decrease in susceptibility of a population to a toxin due to exposure to it [5] while field resistance refers to a control failure under field conditions caused by field-evolved resistance. Genetically engineered *Bt*-crops were first commercialized in 1995. By 2011, *Bt* crops were planted on ca 66 Mha in 25 countries worldwide [3]. The *Bt* crop strategy appeared successful in China and the USA where it had been deployed for control of lepidopteran pests of cotton and corn. Studies reported significant reduction in insecticide use against pests [6–8] and improved biocontrol services [8] associated with *Bt* crops. *Bt* crops suppressed populations of *Helicoverpa armigera* in China, and *Pectinophora* *gossypiella* and *Ostrinia* *nubilalis* in the USA [7,9–11]. Populations of four target pests remain susceptible after more than a decade of intensive *Bt* crop use in the USA, including *Heliothis* *virescens* , *Diatraea* *grandiosella* and the two pests mentioned above [12].

However, despite the success of *Bt* crops, sustainable control of pest populations is threatened by the evolution of resistance [13]. The challenge of deploying efficient management strategies to reduce the risk of rapid evolution of resistance to toxins arises as a major component of the worldwide use of *Bt* crops. In North America, the current success in sustaining susceptibility of some of the major pests is ascribed to the implementation of the “high dose/refuge” insect resistance management (IRM) strategy [12]. This strategy is aimed at delaying the evolution of resistance to *Bt* crops [14] by promoting survival of susceptible insects in refuges of non-*Bt* plants. Among others, a crucial requirement for this strategy is that the toxin is expressed by the plant at such a level that it results in a functional recessiveness of resistant traits. As a consequence, mating between resistant and susceptible individuals should produce functionally susceptible progeny. The assumption of a predominantly recessive inheritance of resistance has been supported by several studies, notably based on laboratory-selected resistance to *Bt* toxins [15,16]. Accordingly, the level of dominance was observed to decrease as the concentration of the toxin increased [17–19]. Moreover, a commonly accepted mechanism of resistance to *Bt* proteins, also called “mode 1” resistance, involves reduced binding of toxin and is therefore expected to result in intrinsically recessive inheritance of resistance [20].

Field resistance has been reported in three lepidopteran species after *Bt*-cotton or *Bt*-corn cultivation: *Spodoptera* *frugiperda* after 4 years in Puerto Rico (leading to the withdrawal of Cry1F *Bt* corn), *Busseola* *fusca* (Fuller) (Noctuidae) after 8 years in South Africa [21], and *P* *. gossypiella* after 6 years in India [22]. In addition, the first report of a coleopteran pest that evolved resistance was recently made for *Diabroticavirgifera* *virgifera* in the USA [23]. Until now, field resistance has been reported to be inherited as an incompletely to highly recessive trait in only one pest, *S* *. frugiperda* [24]. Field-evolved resistance alleles of *H. armigera* in India and *Helicoverpa* *zeae* in the United States have been reported to be semi-dominant [25] and incompletely dominant [4], respectively. Field resistance has largely been ascribed to a lack of sufficient refugia and/or failure to use high-dose *Bt* cultivars [12,26]. However, an ambiguity persists in the “high dose” concept. High dose usually implies that the *Bt* dose is sufficient to kill at least 95% of the insects originating from mating between resistant and susceptible individuals [14]. High-dose failure may thus refer to two interrelated aspects: (i) the level at which the toxin is expressed by the plant, and (ii) the nature of the mechanism involved in the resistance itself. Indeed, the high-dose criterion is operational only when the mechanisms underlying the resistance result in an intrinsically recessive inheritance. When introducing a new *Bt*-trait against a target pest, reliance is therefore largely based on the empirical and indirect criteria of "high-dose" — for example, 25 times the toxin concentration needed to kill 99% of the *Bt*-susceptible larvae in laboratory experiments [27].

To design robust and generic strategies for management of resistance, one has to cope with the potential diversity of evolutionary responses in pest populations [20,28,29]. According to a recent review [5] available precommercialization field data showing that the high-dose standard was not met by *B* *. fusca* could suggest that nonrecessive inheritance of resistance might have hastened its evolution to *Cry 1Ab* in *Bt* corn. Prior to the present study, this prediction remained untested. We further consider the field resistance of *B* *. fusca* in South Africa [21,26,30]. By analyzing the survival of progeny originating from controlled crosses of resistant and susceptible individuals on corn tissue, we intend to bring new insights on the possible diversity of mechanisms involved in resistance to *Bt* crops and their inheritance.

## Materials and Methods

### Ethics statement

The study did not involve endangered or protected species, and no specific permits were required to work with either the plant (corn) or the pest species (stem borer). (i) No special authorization was needed to collect the insect pest in the field (ii). The experiment was carried out with commercially released seeds of corn whose cultivation is unregulated. The permission to collect was systematically obtained from the land owners prior to any field collections. This research was done in North West University entomology laboratories in Potchefstroom, South Africa.

### Experiment

Crosses were performed between an *a priori* susceptible population of *B* *. fusca* originating from Kenya (S0° 24.880 E36°21.587) and a population resistant to the genetically engineered MON810 (with a *Cry1Ab* toxin) originating from South Africa.

*Bt*-resistant larvae (R) were collected from three farms inside the Vaalharts irrigation scheme [30] where resistance is common (farm 1: S27° 49.110 E024°.45.577, farm 2: S27° 79.736 E024°81.434, farm 3: S27° 41.619 E024°42.893). Larvae were collected from late-planted *Bt* corn plants that showed symptoms of stem borer damage. One hundred plants per locality were collected and dissected inside the respective *Bt* corn fields. Approximately 100 larvae per locality were collected and reared in plastic containers (40 x 20 x 15 cm) with aerated lids in the laboratory until the pupal stage. Larvae were provided with freshly cut *Bt* corn stems at 3- to 4-day intervals. Rearing was done under ambient laboratory conditions and a natural day/night photoperiod. The offspring of these field-collected larvae were used in this experiment to perform the crosses. Susceptible insects (S) from Kenya were reared in the laboratory for 3 generations before the experiment was carried out.

The sex of pupae from South Africa and Kenya (F_*0*_) was determined. Individual male and female pupae were assigned identification numbers and placed in 30 mL bottles covered with gauze until adult moths emerged. After emergence, male/female pairs were kept in oviposition chambers following methods described in detail by Kruger et al. [31]. Oviposition chambers measured 30 cm high and 15 cm diameter. The chambers were covered with a fine gauze mesh to prevent escape of moths. A 20-cm-long piece of non-*Bt* corn stem with bases of leaves intact was placed in an upright position in the container. Adults were paired according to three types of crosses: Resistant × Resistant (R × R, 9 pairs), Susceptible × Susceptible (S × S, 23 pairs) and Resistant × Susceptible (R × S, 11 pairs).

Egg batches were carefully removed from each stem at 2-day intervals by cutting off a small piece of the leaf with the egg batch attached to it. Number of eggs per batch was counted and a record kept of the number of eggs laid by each female. The progeny of each cross was split into two groups of approximately 30 larvae each: one group was reared on *Bt* corn stems (MON 810) during the entire experiment and the other was reared on the corresponding non-*Bt* iso-line. The number of larvae varied between 12 and 30 with an average of 27.2 larvae per group. The following two hybrids were used: DKC 78-15B (transgenic, MON810) and CRN 3505 (non-*Bt* near iso-hybrid for DKC 78-15B).

Larvae were reared in glass test tubes (20 x 2.5 cm) each containing a cut corn stem with the base of the whorl still intact. Stems were cut from 4–6-weeks-old potted plants that were grown in a greenhouse. Ten neonate larvae were used per test tube and were placed into the whorl of the cut stem. For the first 16 days freshly cut stems as described above were provided every 2–3 days. Each stem was cut approximately 10 cm below the plant whorl leaving 5 cm of the whorl intact. From day 10 onwards, the number of larvae per test tube was reduced by splitting larvae into separate tubes. From day 16 onwards only a 15-cm-long (10 – 15 mm diameter) cut stem, which was replaced weekly, was provided as food. All tubes were kept in a temperature controlled incubator at 26 ± 1°C. Care was taken to use 1^st^ instar larvae that originated from as many different female moths as possible. In order to describe time course survival in the different crosses, larval survival was recorded every 8 days until pupation (roughly, around 55 days). Pupae were weighed and their sex determined.

### Data analysis

The survival of individuals (number of pupae/initial number of larvae) originating from the different types of crosses R × R, R × S and S × S was plotted as a function of time and further analyzed. All the analyses were performed using the R software [32]. Fisher’s exact tests were performed to compare, (i) the survival of a type of cross among the different types of corn (*Bt* and non-*Bt*), and (ii) the survival between different crosses reared on the same type of corn. In case of multiple comparisons, Bonferroni corrections were applied to minimize the familywise error rate. G-tests were used to assess variation in survival among the families within each treatment.

Estimation of the apparent dominance was provided based on the final survival of larvae (*S*) in the three types of crosses using the following standard formula [33]: *h*_*(S)*_ = (*S*_S×S_ -*S*_R×S_)/(*S*_S×S_ -*S*_R×R_). An approximated distribution of *h*_*(S)*_ was computed using a bootstrap procedure [34] with 1000 iterations (another estimation of dominance based on "mortality phenotypes" is provided in Appendix S1 Figure S1, S2 and S3).

The pupal mass of surviving individuals was compared using a mixed effect Generalized Linear Model [32,35]. This analysis aimed to test the effects of both the three types of crosses and the rearing diet (*Bt* vs non-*Bt* plant). Families of individuals were set as a random factor and a Gaussian family was selected since it corresponded to the best likelihood fit of our data more than Gamma, Log-Normal or other distributions.

## Results

Different types of crosses within and between field-evolved resistant (R) and susceptible (S) strains of *B* *. fusca* were made. Among the R × R, R × S and S × S crosses, respectively 7 out of the 9, 2 out of the 11 and 4 out of the 23 crosses yielded enough viable eggs. The crossing success was not homogeneous among the crosses (*P* = 0.004; Fisher’s exact test); the R × R type performed better than the two others. The low mating success in R × S crosses resulted in susceptible female and resistant male pairs only.

**Figure 1:**
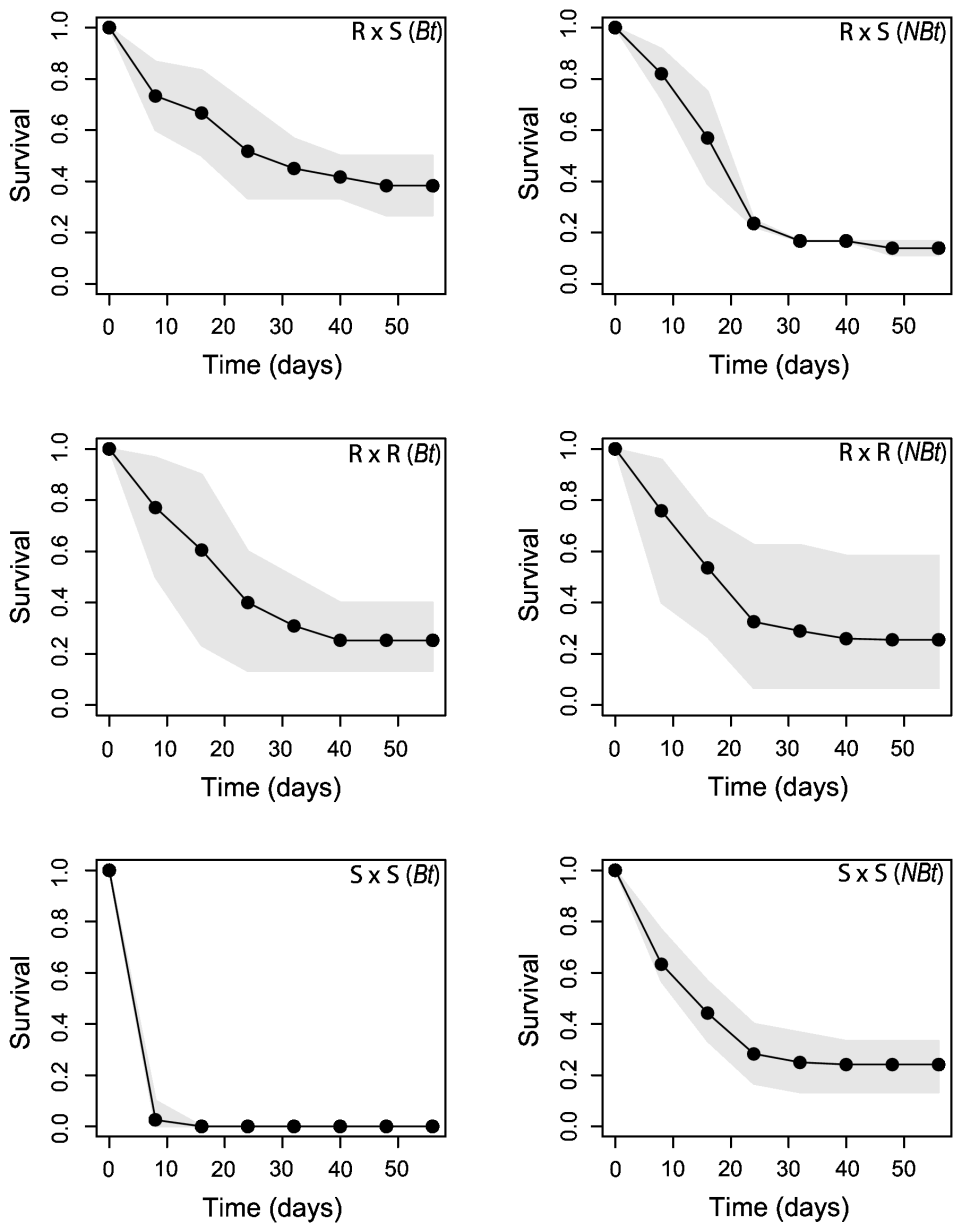
Time course survival of *Busseola fusca* larvae. Larval survival on *Bt* and non-*Bt* (*NBt*) corn as a function of time, for the crosses S × S, R × R and R × S. Points correspond to the average observed survival. Shaded areas correspond to the respective minimum and maximum observed survival.

This experiment showed that the inheritance of resistance traits in *B* *. fusca* reared on *Bt* plant stems was not functionally recessive. In S × S progeny, survival was significantly higher on non-Bt corn (0.242) than on *Bt* corn (0) (*P* < 0.001, Fisher’s exact test), but survival was not significantly higher on non-*Bt* corn than *Bt* corn for either R × R or R × S progeny (Table 1 Figure 1). Resistance of R × S progenies was not compatible with the empirically expected “high dose” criterion for which at least 95% of the R × S progenies should not survive on *Bt* corn. In each R × S progeny the binomial probability to observe an equal or higher level of larval survival under high-dose expectations (95% killed) was close to 0 (*P* < 0.001).

**Table 1:** *Busseola fusca* larval survival in the three crosses (R × R, R × S, and S × S) reared on *Bt* and non-*Bt* corn.

Corn	Cross	*n*	# family	Survival	
*Bt*	S × S	120	4	0.000	A,B,c
*Bt*	R × S	60	2	0.383	A
*Bt*	R × R	195	7	0.246	B
Non-*Bt*	S × S	120	4	0.242	c
Non-*Bt*	R × S	30	2	0.133	
Non-*Bt*	R × R	183	7	0.257	

n: initial number of larvae

A, B, c Identical letters indicate highly significant differences in survival between groups (Fisher’s exact tests), after Bonferroni corrections (*P* ≤ 0.001/9 ; upper case letters denote within corn type differences; lower case letters, differences between corn types for a given cross. The other comparisons were not significantly different (*P* > 0.05/9

Consistent with expectations, the susceptible strain (S × S progeny) reared on *Bt* corn died at an early stage of the experiment. Ninety-eight percent of susceptible larvae (*n* = 120) died within 8 days while the remaining larvae died during the following week. By contrast, survival was > 50% after 16 days in both the R × S and R × R progenies reared on *Bt* corn (Figure 1). No strong disparity appeared in the survival of larvae reared on non-*Bt* corn. We failed to demonstrate any differences among crosses in the survival of larvae reared on non-*Bt* corn (*P* = 0.343, Fisher’s exact test). Nevertheless, after Bonferroni corrections (based on 9 pairwise tests), a marginally significant difference (*P* = 0.016) appeared in the survival of R × S progenies between the two types of corn (Table 1). Variation in survival among families within each of the treatments (i.e., 3 types of crosses × 2 types of corn) was significant only among the R × R progenies reared on non*-Bt* (*P* = 0.005, G-test). The estimation of dominance based on larval survival was *h*_*(S)*_ = 1.560 with a 95% confidence interval of [0.874, 2.566] based on bootstrap procedure.

Moreover, no clear growth inhibition appeared when considering the pupal mass of surviving individuals (Table 2). Besides the expected difference of pupal mass between males and females (*P* = 0.002), no difference according to type of cross (S × S, R × S and R × R) or the rearing diet was observed (*P* > 0.206).

**Table 2:** Pupal mass (g) of *Busseola* *fusca* individuals reared on *Bt* and non-*Bt* corn.

Sex	Cross	*Bt*			Non-*Bt*		
		mean	± *SE*	*n*	mean	± *SE*	*n*
Male	S × S	–	–	0	0.197	0.007	10
Male	R × S	0.196	0.028	10	0.186	0.021	2
Male	R × R	0.197	0.011	16	0.186	0.011	17
Female	S × S	–	–	0	0.222	0.013	8
Female	R × S	0.223	0.012	12	0.201	–	1
Female	R × R	0.249	0.015	22	0.218	0.009	25

*SE*, standard error; n number of pupae.

Males and females originated from the three different types of crosses R × R, R × S, and S × S.

## Discussion

In South Africa, field-evolved resistance in *Busseola* *fusca* resulted in one of the few instances of field resistance reported until now [21]. Notwithstanding the low mating success when performing the crosses, our results showed that the resistance to *Cry 1Ab* in *B* *. fusca* was not recessively inherited, contrary to the important assumptions of the “high dose/refuge” resistance management strategy.

### Specific experimental effects

Our study was based on a laboratory experiment carried out with corn stems; therefore, it did not directly reflect survival on plants in the field. However, the results are consistent with those of a previous study showing resistance of larvae originating from the Valhaarts area in whole plant bioassays conducted in greenhouses [30] and supported by farmers’ perceptions [26]. The two strains used in this study, from Kenya (S) and South Africa (R), are likely to be characterized by differentiated genetic backgrounds. We cannot exclude the fact that genetic differentiation between the two populations might have contributed to the observed differences [36]. However, there was a clear contrast in the response of the different types of crosses, which can be attributed to inherited resistance alleles. We failed to include in our experiment the reciprocal R × S crosses that would otherwise have been necessary to test the existence of maternal effects. It has been shown that maternal effects transmitted by parents that were reared on *Bt*-treated artificial diet could have negative effects on embryogenesis and/or adult fertility of progeny [37]. We cannot exclude that mating between resistant females and susceptible males could result in lower progeny fitness. Reciprocally, our study confirms that high levels of dominance may be reached in *B* *. fusca* without transfer of cytoplasmic content from a resistant female to the progeny, which could increase its fitness [38].

### Evolution of resistance

This study confirmed the prediction of non-recessive resistance based on pre-commercialization data showing that the high-dose standard was not met [5]. *De facto*, functionally non-recessive resistance results in higher proportion of resistant phenotypes in a population as compared to a recessively inherited resistance, all other things being equal. It is thus expected to lead to rapid evolution of resistance in a pest population and to drastically reduce the efficiency of the refuge strategy [4,20]. Simple models [4] suggested that, in such a context, a refuge should account for about 55% of the total surface to delay the time for resistance to develop at 10 years (for an effective dominance ^≈^ 0.8). High dispersal capacities of *B* *. fusca* and an apparent dominant inheritance would be in line with a rapid geographic expansion of resistance in South Africa. Since the first official report of resistance in 2006 (in Christiana), obervations of resistant larvae have been recorded in another area (Valhaarts), about 50 km from the initial site [30].

When the refuge strategy was developed, most of the known cases of pest resistance to *Bt* were strains selected under laboratory conditions. Although Tabashnik et al. [15] highlighted that laboratory resistance could not necessarily predict field resistance, refugia models were mainly based on the assumption of a rather uniformly recessive resistance. Interestingly, among the Lepidoptera species for which field-evolved resistance has been observed and inheritance studied, at least one species ( *B* *. fusca* ) and probably two others (i.e., *H. armigera* [25] and *H* *. zeae* [4]) do not correspond to recessive resistance cases. Moreover, in Chinese populations of *H. armigera* diverse resistance alleles in field-selected populations (including nonrecessive alleles) have been found while no field control failure has been reported [39].

### High dose

A “high-dose” failure may a priori refer to two distinct cases: (i) low-dose-based resistance, which may be intrinsically recessive, and (ii) intrisically non-recessive resistance. In many studies, resistance appeared at least partially recessive at the appropriate doses, killing 100% of susceptible larvae (discriminating doses); at these doses heterozygote individuals for a resistance allele are not expected to survive [40–42]. The underlying causes of a dominant character have been subjected to debate [43–46]. Following Wright’s theory, an intrinsically dominant resistance can be expected when, for example, a pesticide targets the enzymes associated with a multi-step enzymatic pathway [47]. In the case of *Cry* toxins targeting cadherin receptors, recessive or partially recessive inheritance is expected and has been commonly observed. Beyond the observation of a “high dose” failure, our study stresses the need to understand the diversity of field-evolved resistance to develop sustainable resistance management strategies.

To conclude, in South Africa, the case of *B* *. fusca* advocates for anticipated IRM strategies that do not exclude cases of non-recessive resistance. IRM strategies might be complemented by integrated pest management (IPM), and both should be considered together. Recent findings showed that cultivation of *Bt* crops results in reduced insecticide sprays, which could promote biocontrol services in agricultural landscapes. In South Africa, the future emphasis might be to adjust IRM strategies [13] to complement the overall IPM.

## Supporting Information

Appendix S1 **Estimation of the dominance based on **“**mortality phenotypes**”.
(PDF)

Figure S1 *Busseola fusca* larval survival on *Bt* corn as a function of time, for the different crosses, S × S, R × R and R × S. Black dots represent the observed survival in S × S crosses, and the corresponding curve represents the reference model *Φ*_*SS*_. Envelopes correspond to the respective minimum and maximum survival observed among families: hatched envelope encompasses R × R crosses; grey envelope, R × S crosses.
(TIF)

Figure S2 **Proportion of *Busseolafusca* larvae whose mortality phenotype was not compatible with the reference model (*Φ_SS_*) describing the mortality of susceptible larvae over time, in each of the two types of cross R × R (*p*_R×R_ – grey area) and R × S (*p*_R×S_ – transparent area)**.
(TIF)

Figure S3 **Cumulative probability distributions of the two estimations of dominance: *h*_*(S)*_, based on the survival at the end of the experiment (bootstrapping – dotted line) and *h*_*ϕ*_, based on the mortality phenotype (posterior probability – red curve)**.
(TIF)
