# Supplementary material for: Dominant Inheritance of Field-Evolved Resistance to Bt Corn in Busseola fusca
Source: PLoS One. 2013 Jul 2;8(7):e69675. doi: 10.1371/journal.pone.0069675 (PMC3699669; doi:10.1371/journal.pone.0069675)
Supplement: Appendix S1 — Estimation of the dominance based on “mortality phenotypes”. (PDF) [file pone.0069675.s001.pdf]

Appendix S1: estimation of the dominance based on "mortality phenotypes"

A second estimation of dominance was based on the proportion of "mortality phenotypes" (mortality date) that were considered incompatible with the mortality pattern of susceptible individuals. Mortality in the S × S progenies reared on *Bt* was modeled as a function of time using a Weibull distribution characterized by both a shape parameter ($k > 0$) and a scale parameter ($\lambda > 0$). Maximum log-likelihood estimations of those parameters set the reference model (henceforth Φ_{ss}) which describes the mortality dynamics of susceptible individuals on *Bt* corn. Then, on the basis of this model, we computed the probability of individual mortality (in the R × R and R × S progenies) to be incompatible with the mortality phenotype of susceptible individuals (Φss). The density probability associated to the proportion of "non-susceptible" individuals (p – henceforth $p_{\mathrm{R\times S}}$ and $p_{\mathrm{R\times R}}$ relative to the crosses R × S and R × R, respectively) was calculated on the basis of a binomial distribution. The dominance (h_ϕ) was assessed using the following formula: $h_\phi = (p_{\mathrm{S\times S}} - p_{\mathrm{R\times S}}) / (p_{\mathrm{S\times S}} - p_{\mathrm{R\times R}})$, and its associated posterior probability distribution was computed accordingly.

Reference model

The mortality $M(t)$ in the S × S progenies reared on *Bt* was modeled as a function of time using to a Weibull distribution:

$$M(t|k,\lambda) = 1 - e^{-\left(\frac{t}{\lambda}\right)^k} .$$

where $k > 0$ is a shape parameter and $\lambda > 0$ is a scale parameter of the distribution.

Under this model, we considered the probability for an individual *i*, originating from a S × S cross *c*, to die between two observation times T_{ci} and T_{ci}+1. The corresponding likelihood function used to fit the Weibull model was:

$$\ell(k,\lambda)=\prod_{c=1}^{C}\prod_{i=1}^{I}\left[M(T_{ci}+1|k,\lambda)-M\left(T_{ci}|k,\lambda\right)\right]$$

The maximum likelihood estimations of the parameters *k* and *λ* defined the reference model (henceforth, Φ_{ss}) describing the mortality dynamics of susceptible individuals (i.e., originating from S × S) on *Bt* corn.

Proportion of "non-susceptible" phenotypes in the R × R and R × S progeny

Then, on the basis of this model, we discriminated individuals of the R × R and R × S progenies whose observed mortality date was not compatible with the phenotype of susceptible individuals (*Φss*). We classified as "non-susceptible" any individual mortality events that had, conditionally to Φ_{ss}, a probability to be observed lower than 0.001. The following criterion was considered:

$$1-P(O_{ci}|\Phi_{SS})>0.999$$

where O_{ci} is the time interval within which the death an individual R × R or R × S is observed. Individuals surviving up to adult stage were *de facto* considered to meet this criterion.

The density probability of the proportion of individuals meeting this criterion in each cross (*p* – henceforth $p_{R\times S}$ and $p_{R\times R}$ relative to the crosses R × S and R × R, respectively) was calculated on the basis of a binomial distribution:

$$\Psi_c\left(p|n_c,N_c\right)=C_{N_c}^{n_c}\,p^{n_c}(1-p)^{n_c}$$

where n_c denotes the observed number of individuals which were discriminated as non-compatible with the Φ_{ss} model out of the N_c individuals reared on *Bt*.

The dominance corresponding to those distributions was assessed using the same formula as before: $h_\phi = (p_{S\times S} - p_{R\times S}) / (p_{S\times S} - p_{R\times R})$, and the associated posterior probability distribution was computed accordingly.

Results

The maximum log-likelihood fit (-14.02) of the reference Weibull model (Φ_{ss}) was obtained with the parameter values $k = 2.511$ and $\lambda = 0.595$ (Figure S1). The maximum log-likelihood estimation of the proportions of individuals which did not die according to the modeled pattern of susceptible insects ($p_{R\times S}$ and $p_{R\times R}$) were comparable: $p_{R\times S} = 0.667$ and $p_{R\times R} = 0.610$. The interval defining 95% of their probability distribution, [0.539, 0.772] in R × S and [0.540, 0.676] in R × R overlapped (Figure S2). The maximum likelihood estimation of the dominance based on the mortality phenotype was close to 1, $h_\phi = 1.088$ with a 95%-interval of [0.854 – 1.327], while the dominance estimation based on larvae survival at the end of the experiment was higher, $h_S = 1.560$ with a 95%-interval [0.874, 2.566] (Figure S3).
