# Supplementary figures and images for: Dominant Inheritance of Field-Evolved Resistance to Bt Corn in Busseola fusca
Source: PLoS One. 2013 Jul 2;8(7):e69675. doi: 10.1371/journal.pone.0069675 (PMC3699669; doi:10.1371/journal.pone.0069675)

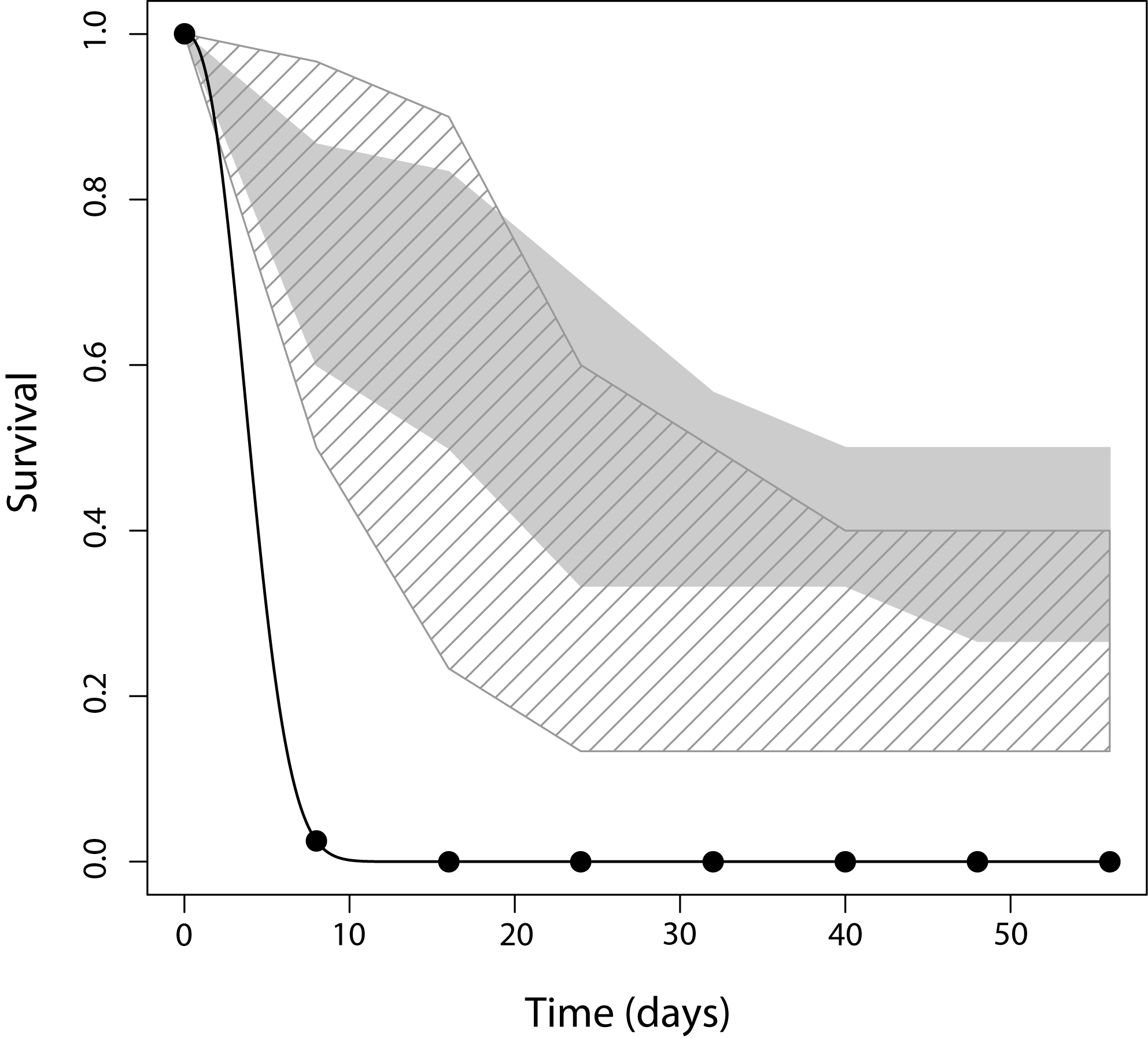

Supplement: Figure S1 — Black dots represent the observed survival in S × S crosses, and the corresponding curve represents the reference model Φ SS. Envelopes correspond to the respective minimum and maximum survival observed among families: hatched envelope encompasses R × R crosses; grey envelope, R × S crosses. (TIF) [file pone.0069675.s002.tif]

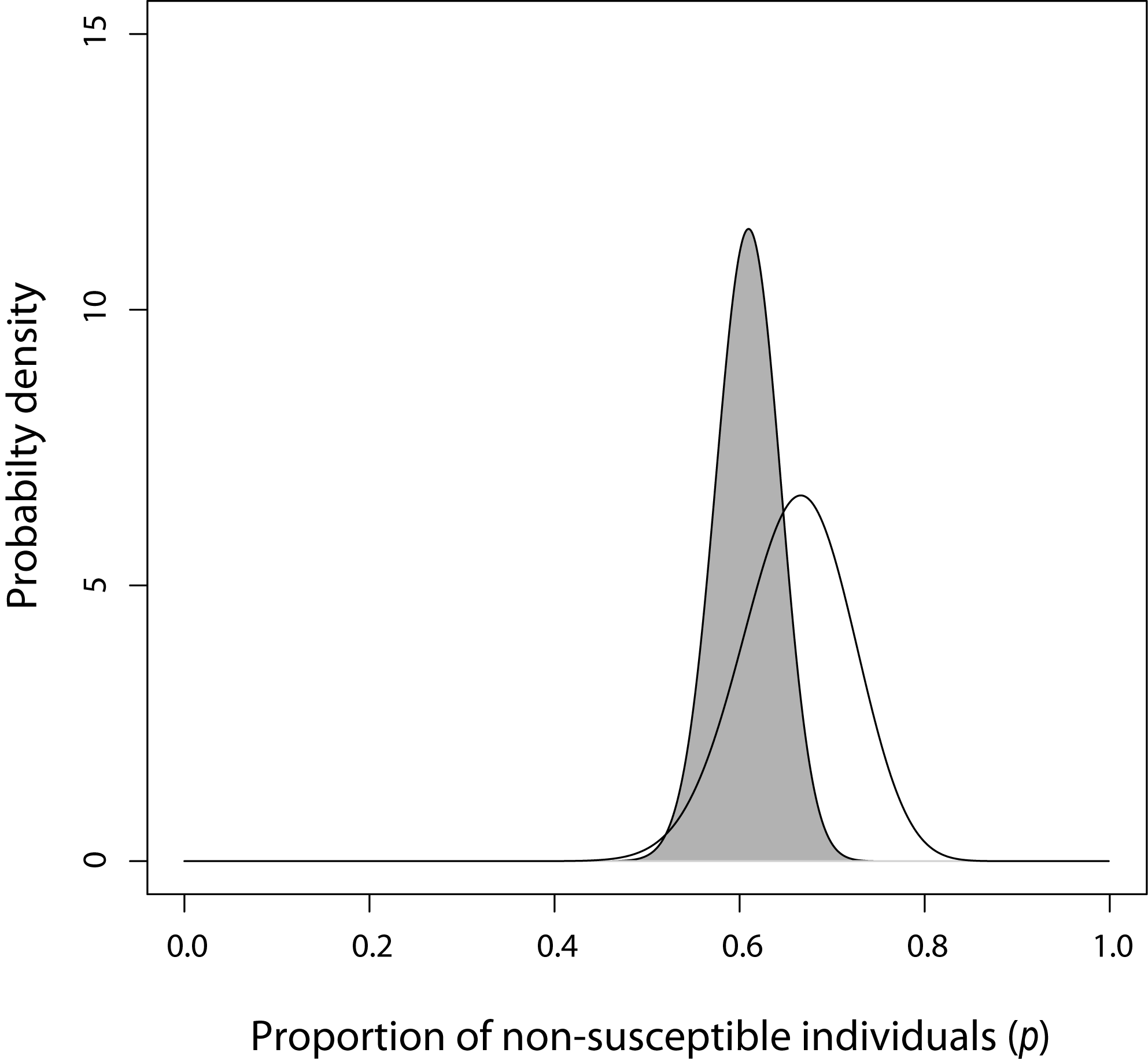

Supplement: Figure S2 — Proportion of Busseolafusca larvae whose mortality phenotype was not compatible with the reference model (ΦSS) describing the mortality of susceptible larvae over time, in each of the two types of cross R × R (pR×R – grey area) and R × S (pR×S – transparent area). (TIF) [file pone.0069675.s003.tif]

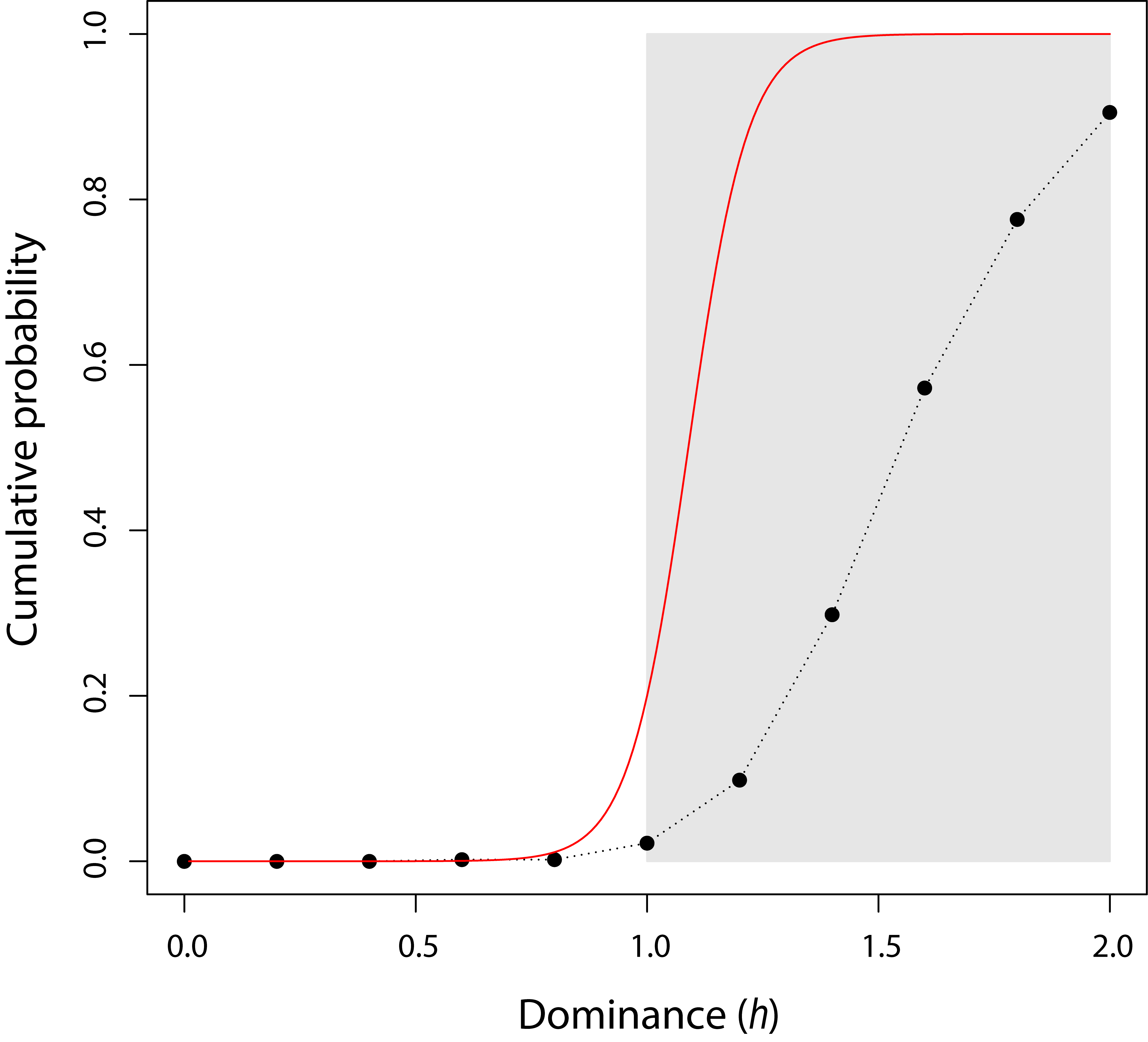

Supplement: Figure S3 — Cumulative probability distributions of the two estimations of dominance: h(S), based on the survival at the end of the experiment (bootstrapping – dotted line) and hϕ, based on the mortality phenotype (posterior probability – red curve). (TIF) [file pone.0069675.s004.tif]
